# Pyrethroids and organophosphate resistance in *Aedes aegypti* (Diptera: Culicidae) and their underlying mechanisms

**DOI:** 10.1186/s13071-026-07252-0

**Published:** 2026-02-07

**Authors:** Wan Fatma Zuharah, Shao-Hung Dennis Lee, Fatin Nabila Abdullah, Asfa Nurizzah Zin Azman, Ikhsan Guswenrivo, Beni Ernawan, Titik Kartika, Theerakamol Pengsakul, Tianyun Su, Chow-Yang Lee

**Affiliations:** 1 Medical Entomology Laboratory, School of Biological Sciences, Universiti Sains Malaysia, 11800 Minden, Penang Malaysia; 2 Department of Entomology, University of California, 900 University Ave, Riverside, CA 92521 USA; 3 Research Center for Public Health and Nutrition, National Research and Innovation Agency of the Republic of Indonesia (BRIN), Cibinong, Bogor 16911 Indonesia; 4 Research Center for Applied Zoology, National Research and Innovation Agency of the Republic of Indonesia (BRIN), Cibinong, Bogor 16911 Indonesia; 5 Faculty of Environmental Management, Prince of Songkhla University, Hat Yai, Songkhla, 90110 Thailand; 6 EcoZone International LLC, Riverside, CA 92506 USA

**Keywords:** *Aedes aegypti*, *Kdr* mutation, Mosquitoes, Metabolic enzyme, Resistance

## Abstract

**Background:**

For decades, insecticides have been central to controlling the yellow fever mosquito, *Aedes aegypti* (L.), but extensive use has driven resistance development. This study investigates resistance of *Ae. aegypti* to pyrethroids (permethrin, deltamethrin) and organophosphates (malathion, pirimiphos-methyl) and their underlying mechanisms across Malaysia, Thailand, Indonesia, and the USA.

**Methods:**

Adult female *Ae. aegypti* (3–5 days old, non-blood-fed) were subjected to World Health Organization (WHO) tube bioassays using 0.4% permethrin, 0.03% deltamethrin, 5% malathion, and 60 mg/m^2^ pirimiphos-methyl. Each assay included four replicates of 25 mosquitoes, with mortality assessed at 24 h post-exposure. Genomic DNA was extracted from 10 resistant individuals per population, and two coding regions of the voltage-gated sodium channel (VGSC) gene (domains II and III) were amplified and sequenced to detect known and novel *kdr* mutations. For biochemical analysis, 40 newly emerged, non-blood-fed females per strain were individually homogenized to quantify mixed-function oxidase (MFO), esterase (α- and β-EST), glutathione S-transferase (GST), and acetylcholinesterase (AChE) activity.

**Results:**

High resistance levels were recorded in Malaysian and US *Ae. aegypti* strains, with low mortality ranging between 9% and 22% for pyrethroids. New mutations T1520I (8–15%) and I1011M (10–15%) were identified in Malaysian populations, the first detection of T1520I in the country, while V1016I (10%) was newly detected in Indonesian strains. Malaysian mosquitoes had multiple *kdr* mutations (S989P, V1016G, F1534C, and T1520I) in triple- and quadruple-haplotype combinations. The US Riverside strain showed a nine- to 10-fold increase in β-EST and three- to fivefold increase in MFO and GST activity compared to the VCRU susceptible strain, indicating strong metabolic resistance. In contrast, the highly resistant Malaysian Hamna strain exhibited no significant upregulation (*P* > 0.05) in detoxifying enzymes, suggesting resistance was driven primarily by *kdr* mutations. Thai strains lacked *kdr* mutations but exhibited altered AChE (20–35% remaining activity) and elevated GST (2–3 times higher than control).

**Conclusions:**

The detection of novel *kdr* mutations and diverse resistance mechanisms underscores the adaptability of *Ae. aegypti* to insecticide pressure and highlights the urgent need for continuous monitoring and integrated resistance management strategies.

**Graphical Abstract:**

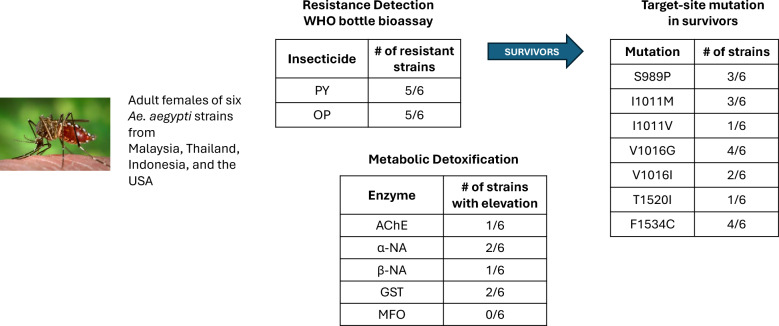

## Background

The yellow fever mosquito *Aedes aegypti* is a globally distributed species and an important vector of the viral pathogens of yellow fever, dengue, chikungunya, and Zika infections [1]. This species has exhibited dynamic invasive distribution patterns worldwide. In Southeast Asia, the species’ expansion since the nineteenth century has been linked to maritime trade, with higher prevalence near coastal settlements [2]. While tropical and subtropical regions remain favorable due to suitable climate conditions, human influence and artificial environments also contribute to maintaining this species’ range in temperate and subtropical areas [3]. In the United States, *Ae. aegypti* was reported in 183 counties across 26 states from 1995 to 2016, with a persistent presence in 94 counties [4]. Genetic analyses revealed 20 distinct haplotypes across the Americas, Africa, and Asia, suggesting multiple introductions and complex phylogeographical structures [5]. Local shifts in distribution, such as in southern Florida, may be due to interspecific interactions with *Aedes albopictus* [6].

Resistance in *Ae. aegypti* varies by location and is influenced by local insecticide use and different resistance mechanisms. It develops through target-site mutations, increased metabolic detoxification, and behavioral avoidance [7–9]. These adaptations reduce the effectiveness of insecticides, making control efforts more difficult and highlighting the need for ongoing surveillance [10, 11]. Target-site resistance, caused by mutations in the *para* sodium channel gene, can reduce the binding affinity of insecticides, making them less effective [12], especially against pyrethroids (PYs) [13, 14]. In Southeast Asia, *Ae. aegypti* populations have shown widespread resistance to PYs [15–17], as well as other insecticide classes such as organophosphates (OPs) and carbamates, particularly in Thailand and Malaysia [18].

The S989P mutation in domain II of the voltage-gated sodium channel (VGSC) often occurs with V1016G in deltamethrin-resistant populations [19], while other mutations like V1023G and S996P usually combine with increased monooxygenase activity to enhance resistance [20]. Recent research in Malaysia identified a new knockdown resistance (*kdr*) mutation, A1007G, in the VGSC of *Ae. aegypti* mosquitoes [21, 22]. Additionally, three common *kdr* mutations, S989P, V1016G, and F1534C, were found in *Ae. aegypti* from multiple states, including Selangor, Penang, and Kelantan [21, 22]. Similar mutation patterns have been observed in Indonesia [23, 24], although mutations in the acetylcholinesterase (AChE) gene have not been reported there [25]. In Thailand, *Ae. aegypti* mosquitoes frequently carry S989P, and the heterozygous S989P+V1016G+F1534C (triple-mutant) genotype offers high resistance, significantly reducing the effectiveness of thermal fogging. V1016G is widespread in Thai populations and is strongly linked to deltamethrin resistance, while F1534 is associated with permethrin resistance [26, 27]. Meanwhile, high levels of the V1016I and F1534C *kdr* mutations, related to PY resistance, have been repeatedly documented in Florida, USA, since 2018 [28].

Enhanced metabolic detoxification is another important resistance mechanism. This involves increased activity of enzymes that break down insecticides, making them less effective [15, 18]. Metabolic resistance may include elevated levels of cytochrome P450 enzymes, which are crucial for breaking down toxic substances, further complicating vector control efforts [29]. This increased enzyme activity is a key factor in metabolic resistance to insecticides like deltamethrin and malathion [30].

This study evaluated the resistance profiles of *Ae. aegypti* from Malaysia, Indonesia, Thailand, and the USA, representing areas with different levels of arboviral endemicity. The goal was to understand the roles of VGSC mutations and metabolic enzyme activity in resistance to pyrethroids (PYs) and organophosphates (OPs). By analyzing VGSC mutations and enzyme activity linked to resistance, these findings can support locally tailored vector control strategies. The data offer evidence-based guidance for optimizing insecticide choice, enhancing rotation or combination strategies, and developing regional resistance management policies to maintain long-term control effectiveness.

## Methods

### Mosquitoes

Field sampling of *Ae. aegypti* mosquitoes was carried out using ovitraps, consisting of black tin cans (height 10.4 cm, diameter 7.0 cm). A hardboard paddle (3 cm × 14.7 cm × 0.3 cm) was placed vertically inside each ovitrap, with the textured surface facing upward to encourage oviposition. Dechlorinated tap water was added to each receptacle to a depth of 6.0 cm, using 200 ml of dechlorinated tap water. The ovitraps were set up at the sampling site for at least 5 days to promote oviposition by adult female *Ae. aegypti* mosquitoes.

**Fig. 1:**
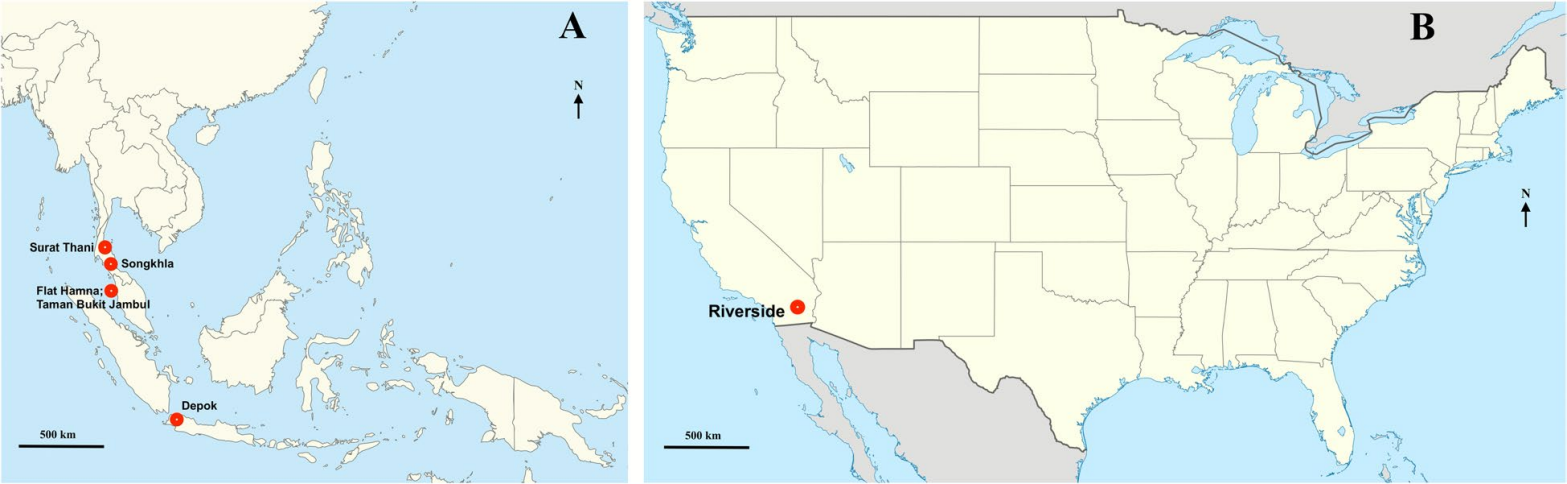
Location of the sampling sites for *Ae. aegypti* collection, which were located in **A** Malaysia (Flat Hamna and Taman Bukit Jambul, Penang), Indonesia (Depok), Thailand (Songkhla and Surat Thani), and **B** the USA (Riverside, CA). Map source: Wikimedia Commons

Mosquitoes used in this study were collected from Malaysia, specifically at Flat Hamna (FH) (5.351133, 100.3016) and the Taman Bukit Jambul Apartment (TBJ) in Penang (5.336123, 100.287673), as well as from one site in Depok (DP), West Java Province, Indonesia (6.2338, 106.4921), and two sites in Thailand, Songkhla (SK) (7.1222, 100.3548) and Surat Thani (ST) (9.128875, 99.325629) (Fig. 1A). Additionally, one strain from Riverside (RS), California (33.918933, −117.372186), was selected from North America (Fig. 1B). The mosquitoes were collected using 40 ovitraps filled with water and containing paddles as oviposition substrates. These traps were left at the study sites for 5 days before being brought back to the lab for species identification and culture. All mosquitoes were collected between April 2023 and December 2023, except for the RS strain, which was collected in July 2022.

The collected larvae were transported to the laboratory, where they were cultured in enamel trays filled with dechlorinated tap water. The culture conditions were controlled to maintain a temperature of (28 ± 2) °C, relative humidity of 70–85%, and a photoperiod of 12 h/12 h light/darkness. The larvae were fed a mixture of dog biscuit, beef liver, yeast, and milk powder, prepared in a 2:1:1:1 weight ratio as a fine powder at a dose of 1 g/day. The larvae were allowed to develop into adults, labeled as the F_0_ generation. If the F_0_ population was too small for a bioassay, they were bred to produce F_1_ and *F*_2_ generations. The VCRU (Vector Control Research Unit) strain served as the susceptible reference strain for comparison with other field strains. This strain has been cultured since the 1960s, over more than 345 generations, maintained by the Vector Control Research Unit (Universiti Sains Malaysia, Penang). It has been reared under insecticide-free conditions for more than 30 years.

### Adult mosquito bioassays

Adult bioassays were conducted using the standardized protocol established by the World Health Organization (WHO) [31]. The study used non-blood-fed adult female mosquitoes of the field strains against PY and OP insecticides in four replicates, each comprising 25 non-blood-fed female mosquitoes aged 3–5 days. Susceptibility tests on *Ae. aegypti* were performed using the WHO-impregnated papers at discriminating concentrations for PYs (0.4% permethrin and 0.03% deltamethrin) and OPs (60 mg/m^2^ pirimiphos-methyl and 5% malathion). Controls were conducted in duplicate for each insecticide, employing silicone oil for PY control and olive oil for OP control.

A total of 25 adult female *Ae. aegypti* mosquitoes were placed in holding tubes and acclimatized for 1 h. Following this acclimation interval, any damaged, injured, or dead mosquitoes were replaced with healthy individuals. Subsequently, the mosquitoes were transferred from the holding tube to a test tube containing insecticide-impregnated paper. Data pertaining to knockdown effects were systematically recorded at 5-min intervals over a period of 1 h of exposure to the insecticides. At the end of the exposure period, the mosquitoes were promptly returned to the holding tubes, where they were provided with cotton wool moistened with a 10% sucrose solution as an energy source. The mortality rate was subsequently assessed 24 h post-exposure. The test was conducted at a temperature of 28 ± 2 °C, relative humidity of 78 ± 10%, and with a 12:12 light/dark photoperiod.

### Statistical test for susceptibility of *Ae. aegypti*

The percentage mortality observed at 24 h after exposing adult mosquitoes to insecticides in bioassay experiments was used to assess their susceptibility status according to the criteria established by WHO [31]. Mosquitoes are classified as (1) susceptible if the mortality rate is between 98% and 100%, (2) incipiently resistant if it falls between 90% and 97%, and (3) resistant if it is less than 90% [31]. If the control mosquito mortality exceeded 20%, the experimental data were discarded. Conversely, when control mortality was between 5% and 20%, the mortality percentage was adjusted using Abbot’s formula [32]:

$$= \frac{{{{\% Treated}}\;{\text{ mortality}} - {\text{ \% Control}}\;{\text{ mortality }}}}{{100 - {{\% Control }}\;{\mathrm{mortality}}}} \times 100\%.$$

In the present study, Abbot’s formula was not applied due to the 0% mortality. The 24-h mortality data were subjected to a one-way analysis of variance (ANOVA) to determine significant differences between localities for each insecticide using SPSS version 28.0. Data were tested for homogeneity and normality before the test. The transformation was performed using a log transformation to fulfill the assumption of one-way ANOVA.

### Knockdown resistance (*kdr*) mutations of the VGSC gene

Following the WHO bioassays, we selected the surviving insects (24 h post-treatment) from each site × insecticide stratum for genotyping, with up to 10 resistant survivors selected for sequencing. The final number of genotyped specimens varied among strains due to differences in the number of survivors. In total, 188 individual mosquitoes were genotyped across domains II (DIIS6) and III (DIIIS6) of the VGSC; per-site and per-insecticide sample sizes are provided in Table 1. DNA was extracted from 10 specimens of live *Ae. aegypti* mosquitoes that exhibited resistance after being tested with PY and OP insecticides, using the DNeasy extraction kit (Qiagen, Germany; cat. no. 69506) following the manufacturer’s protocol. The DNA concentration and purity were measured using a NanoDrop spectrophotometer (Thermo Fisher Scientific). To ascertain *kdr* mutations, two segments of the coding region of the VGSC gene, encompassing exons 19 through 31 (including the 989, 1011, 1016, 1007, and 1534 coding positions), were amplified from DNA samples and subsequently subjected to Sanger sequencing. A polymerase chain reaction (PCR) mixture totaling 25 μl was formulated utilizing Platinum SuperFi II PCR Master Mix. Reactions (25 µl) contained 12.5 µl of master mix, 0.2 µM of each primer, 10–50 ng of DNA template (extracted with DNeasy Blood & Tissue Kit, Qiagen), and nuclease-free water to volume.

**Table 1:** Distribution of single and multiple loci of the genotypic combination in domains II and III of the VGSC gene in *Ae. aegypti* from Malaysia, Thailand, Indonesia, and the United States of America

Sites	Insecticides	Phenotype	N*	Domain II														
				S989P			I1011M			11011 V			V1016G			V1016I		
				SS	SP	PP	II	IM	MM	II	IV	VV	VV	VG	GG	VV	VG	II
Hamna Flat, Malaysia	5% Malathion	Alive	10	0	0	0	0	1	0	0	0	0	0	0	0	0	0	0
Taman Bukit Jambul, Malaysia		Alive	2	0	0	2	0	0	0	0	0	0	0	0	2	0	0	0
Songkhla, Thailand		Alive*	3															
Surat Thani, Thailand		Alive*	10															
Depok, Indonesia		None**	0															
Riverside, California, USA		None**	0															
Hamna Flat, Malaysia	60 mg/m^2^ pirimiphos-methyl	Alive	10	0	2	0	0	0	0	0	0	0	0	2	0	0	0	0
Taman Bukit Jambul, Malaysia		Alive	10	0	0	4	0	0	0	0	0	0	0	2	3	0	0	0
Songkhla, Thailand		Alive	10	0	0	0	0	0	1	0	0	0	0	0	0	0	0	0
Surat Thani, Thailand		Alive*	10															
Depok, Indonesia		None**	10															
Riverside, California, USA		Alive	10	0	0	0	0	0	4	0	0	0	0	0	0	0	2	2
Hamna Flat, Malaysia	0.4% permethrin	Alive*	10															
Taman Bukit Jambul, Malaysia		Alive	10	0	0	2	0	0	0	0	0	0	0	0	0	0	0	0
Songkhla, Thailand		Alive*	3															
Surat Thani, Thailand		Alive*	10															
Depok, Indonesia		Alive	10	0	1	1	0	0	0	0	0	0	0	0	0	0	0	3
Riverside, California, USA		Alive	10	0	0	0	0	0	0	0	0	0	0	0	3	0	0	0
Hamna Flat, Malaysia	0.03% deltamethrin	Alive	10	0	0	2	0	0	0	0	0	0	0	0	2	0	0	0
Taman Bukit Jambul, Malaysia		Alive	10	0	0	1	0	0	0	0	0	2	0	0	0	0	0	0
Songkhla, Thailand		Alive*	10															
Surat Thani, Thailand		Alive	10	0	0	0	0	0	0	0	0	0	0	0	0	0	0	0
Depok, Indonesia		Alive	10	0	0	1	0	0	0	0	0	0	0	4	0	0	0	0
Riverside, California, USA		Alive	10	0	0	0	0	0	0	0	0	0	0	0	0	0	0	7

Sites				Domain III						Domain II and III							
				T1520I			F1534C			Type 1	Type 2	Type 3	Type 4	Type 5	Type 6	Type 7	Type 8
				TT	IT	II	FF	FC	C C	S989P/V1016G	S989P/T1520I	I1011V/F1534C	V1016G/T1520I	V1016I/F1534C	T1520I/F1534C	S989P/V1016G/F1534C	S989P/V1016G/T1520I/F1534C
Hamna Flat, Malaysia	5% Malathion	Alive	10	0	0	0	0	0	1	0	0	0	0	0	0	0	0
Taman Bukit Jambul, Malaysia		Alive	2	0	0	0	0	0	1	1	0	0	0	0	0	1	0
Songkhla, Thailand		Alive	3														
Surat Thani, Thailand		Alive	10														
Depok, Indonesia		None	0														
Riverside, California, USA		None	0														
Hamna Flat, Malaysia	60 mg/m^2^ pirimiphos-methyl	Alive	10	0	0	0	0	0	4	2	0	0	0	0	0	0	0
Taman Bukit Jambul, Malaysia		Alive	10	0	0	2	0	0	1	0	1	0	1	0	0	0	1
Songkhla, Thailand		Alive	10	0	0	0	0	0	0	0	0	0	0	0	0	0	0
Surat Thani, Thailand		Alive	10														
Depok, Indonesia		None	10														
Riverside, California, USA		Alive	10	0	0	0	0	0	6	0	0	0	0	1	0	0	0
Hamna Flat, Malaysia	0.4% permethrin	Alive	10														
Taman Bukit Jambul, Malaysia		Alive	3	0	0	5	0	0	5	2	0	0	0	0	5	0	0
Songkhla, Thailand		Alive	10														
Surat Thani, Thailand		Alive	10														
Depok, Indonesia		Alive	10	0	0	0	0	0	0	2	0	0	0	0	0	0	0
Riverside, California, USA		Alive	10	0	0	0	0	0	10	0	0	0	0	3	0	0	0
Hamna Flat, Malaysia	0.03% deltamethrin	Alive	10	0	0	0	0	1	0	2	0	0	0	0	0	0	0
Taman Bukit Jambul, Malaysia		Alive	10	0	0	1	0	0	1	0	0	1	0	0	0	0	0
Songkhla, Thailand		Alive	10														
Surat Thani, Thailand		Alive	10	0	0	0	0	0	0	0	0	0	0	0	0	0	0
Depok, Indonesia		Alive	10	0	0	0	0	0	0	1	0	0	0	0	0	0	0
Riverside, California, USA		Alive	10	0	0	0	0	0	0	0	0	0	0	5	0	0	0

*No mutations were detected in survivor mosquitoes. ** No survivors available for resistance analysis as all tested mosquitoes were dead

Genotype abbreviations: SS**,** FF**,** VV**,** TT**,** II = homozygous susceptible; PP**,** GG**,** CC**,** MM = homozygous resistant; and combinations such as SP**,** FG**,** VC**,** TI**,** IM** = **heterozygous genotypes containing one wild-type and one mutant allele

A total of two sets of PCR reactions were prepared for each DNA specimen for (1) domain II (which includes mutations at S989P, A1007G, I1011M/V, L1014F, V1016G/I, and T1520I), where the initial amplification of fragments was conducted utilizing primers AaSCF1 (AGACAATGTGGATCGCTTCC) and AaSCR4 (GGACGCAATCTGGCTTGTTA), and for (2) domain III, where primers AaSCF7 (GAGAACTCGCCGATGAACTT) and AaSCR7 (GACGACGAAATCGAACAGGT) were employed to identify the F1534C mutation [19]. Expected amplicon size was 480 base pairs (bp) for domain II and 740 bp for domain III. The PCR reactions were carried out with an initial denaturation temperature of 98 °C for 30 s, followed by 30 cycles consisting of denaturation at 98 °C for 15 s, annealing at 60 °C for 30 s, and extension at 72 °C for 1 min. This ended in a final elongation phase at 72 °C for 10 min and was subsequently maintained at 4 °C.

The PCR products were subjected to size-based separation on a 1.1% agarose gel. The gel-containing PCR products were stained with 1 µl Bio-Rad UView 6× loading dye and run at 140 V for 40 min in Tris-acetate-ethylenediaminetetraacetic acid [EDTA] (TAE) buffer. The results were visualized under ultraviolet light. The PCR products were purified using ExoSAP at a 5:2 ratio and sent to Retrogen, Inc. (San Diego, CA, USA) for sequencing. The DNA sequencing process was completed with primers AaSCF3 (GTGGAACTTCACCGACTTCA) and AaSCR6 (CGACTTGATCCAGTTTGGAGA) for domain II and AaSCR8 (TAGCTTTCAGCGGCTTCTTC) for domain III of *Ae. aegypti* [19]. Sequencing data obtained from Retrogen were aligned using ClustalW, and protein sequences were translated using MEGA v12. Heterozygous genotypes were identified as mixed base calls (overlapping peaks) in chromatograms, verified by bidirectional sequencing and alignment in MEGA v12. Only chromatograms with Phred (Phil's Read Editor) scores ≥ 30 and consistent peak patterns across both strands were retained. The six unique DNA haplotype sequences were deposited in the GenBank database under accession numbers PX559884–PX559849.

### Quantification of metabolic detoxification enzymes

The experimental assays were performed on newly emerged F_0_ generation female mosquitoes (within 24 h) of *Ae. aegypti* collected from Asian countries and the USA, which were obtained through field collection methods described in the previous section. The quantification of metabolic enzymes was established using a standard protocol for assessing metabolic resistance described by WHO [33] and Valle et al. [34]. The chemicals used for the biochemical assays were as follows: α-naphthyl acetate (≥ 98%, Sigma-Aldrich, St. Louis, MO, USA), α-naphthol (≥ 99%, Spectrum Chemical Mfg. Corp., Gardena, CA, USA), β-naphthol (≥ 99%, Spectrum Chemical Mfg. Corp.), β-naphthyl acetate (≥ 98%, Sigma-Aldrich), p-nitrophenyl acetate (≥ 98%, Sigma-Aldrich), 1-chloro-2,4-dinitrobenzene (CDNB) (99%, Acros Organics, Carlsbad, CA, USA), fast blue B salt (MP Biomedicals, LLC, Irvine CA, USA), and reduced glutathione (GSH) (99%, Chem-Impex International, Inc., Wood Dale, IL, USA).

#### Homogenization of *Ae. aegypti*

Forty adult *Ae. aegypti* individuals from each locality, including the VCRU susceptible strain, collected on the first day of emergence without a blood meal were selected for biochemical assays. Each mosquito was homogenized using a mortar and pestle in 300 µl of distilled water and kept on ice to reduce proteolysis. For the reference strain, VCRU, 40 individuals underwent homogenization. Un-centrifuged aliquots of 25 µl and 20 µl were used to measure AChE and mixed-function oxidase (MFO), respectively. The remaining aliquots were centrifuged at 12,000×*g* for 60 s. The supernatant was collected for assays of esterase (EST), glutathione S-transferase (GST), and total protein.

#### MFO (monooxygenase) titration assay

The assay was carried out based on the methodology described by WHO [33]. To summarize, 20 μl of microfuge supernatant was mixed with 60 μl of 0.625 M potassium phosphate buffer (pH 7.2) and 200 μl of tetramethylbenzidine (TMBZ) solution (0.012 g 3,3,5,5-tetramethylbenzidine + 6 ml methanol + 18 ml sodium acetate buffer 250 mM, pH 5.0). After adding 25 μl of 3% hydrogen peroxide, the mixture was left to sit at room temperature in darkness for 90 min.

MFO or monooxygenase activity was quantified according to the WHO standard protocol using TMBZ as a chromogenic substrate. Absorbance was measured at 650 nm, and enzyme activity was expressed as equivalent cytochrome P450 content per minute per milligram of total protein (equivalent cytochrome P450/min/mg protein). The “equivalent cytochrome P450” refers to the conversion of reaction products of hemoproteins including P450 to concentrations based on a standard curve of cytochrome c (Sigma-Aldrich, C3131). This conversion allows for comparison of relative P450 monooxygenase content across mosquito strains. Equivalent cytochrome P450/min/mg protein was used to measure enzymatic activity using an Epoch 2 microplate spectrophotometer (BioTek Instruments, Inc., Winooski, VT, USA). Three positive controls were run using 20 μl of cytochrome c in place of mosquito homogenate, and three negative controls were performed using 20 μl of 0.625 M potassium phosphate buffer (pH 7.2).

#### Altered AChE assay

The activity of AChE was assessed either with or without the propoxur inhibitor present, labeled as AChE and AChI, using two separate 96-well plates. A total of 145 μl of Triton/Na phosphate (prepared with 5 ml of 100% Triton X-100 in 50 ml of 1 M sodium phosphate buffer at pH 7.8 and 455 ml of distilled water) and 10 μl of 5,5′-dithiobis-(2-nitrobenzoic acid)(DTNB)/Na phosphate (prepared before use with 10 mM DTNB in 100 mM sodium phosphate buffer at pH 7.0) were added to 25 μl of mosquito homogenate prepared in duplicate.

Each well in the AChE plates contained 10 mM acetylcholine iodide in grade water without propoxur. Propoxur (6 μl of 0.1 M in acetone) was applied to the AChI plates in addition to 10 mM acetylcholine iodide in grade water. Both AChE and AChI plates were incubated for 1 h at room temperature and were read at 405 nm using an Epoch 2 microplate spectrophotometer (BioTek Instruments, Inc., Winooski, VT, USA). Three negative and three positive controls were provided by replacing the mosquito homogenate with 25 μl of sterile distilled water only. The findings were presented as a percentage of the remaining activity in both the inhibited control and the inhibited fraction.

#### EST assays

For EST assays, first, 10 μl of the mosquito homogenate supernatant was applied to each well in duplicate. Then, 200 μl of 30 mM α-naphthyl acetate was added to one set of samples, while 200 μl of 30 mM β-naphthyl acetate was added to the other set. The plate was incubated at room temperature for 15 min. After incubation, each well was filled with 50 μl of fast blue stain, and the mixture was left to incubate for an additional 5 min. The reaction was read at 570 nm using an Epoch 2 Microplate Spectrophotometer (BioTek Instruments Inc., Winooski, VT, USA).

Three positive controls were conducted using 10 μl of α-naphthol at 0.5 μg/ml, while three negative controls were performed using 10 μl of sterile distilled water for the α-EST assay. Similarly, three negative controls were performed with 10 μl of sterile distilled water for the β-EST test, and three positive controls were performed with 10 μl of 0.5 μg/ml β-naphthol. EST activity for each substrate was calculated using standard absorbance curves corresponding to known concentrations of α–naphthol or β–naphthol. Enzymatic activity was expressed as nmol of α-naphthol/min/mg protein or nmol β-naphthol/min/mg protein.

#### GST assay

A total of 15 μl of mosquito homogenate was applied to each microtiter plate well in two test duplicates; 195 μl of a working solution of CDNB was used to measure GST activity. This working solution, which was prepared by combining 0.0615 g of reduced GSH in 20 ml of 100 mM potassium phosphate buffer at pH 6.5 with 0.0042 g CDNB diluted in 1 ml of methanol, was administered to each duplicate of the mosquito homogenate. Three positive and negative controls were prepared using 15 μl of sterile distilled water. The absorbance was read at 340 nm every minute for 20 min using an Epoch 2 microplate spectrophotometer (BioTek Instruments, Inc., Winooski, VT, USA). The GST activity was calculated by converting the data using Beer’s law (*A* = *εcl*), with a path length of 0.6 cm and an extinction coefficient of 4.29 µM^−1^.

#### Total protein assay

All enzyme activity assays were normalized using protein concentration as a correction factor to account for individual mosquito size variations. A commercial protein assay kit (Bio-Rad, USA) was used to generate the bovine serum albumin (BSA) standard curve, which was then used to convert and calculate the protein concentration for each sample.

After centrifugation, 10-μl duplicates of mosquito homogenates were plated in 96-well plates. To perform the protein test, 10 μl of mosquito homogenate was mixed with 300 μl of diluted Bio-Rad dye reagent. This mixture was then incubated at room temperature for 3 to 5 min. For negative controls, three wells containing 10 μl of distilled water were used, while three wells containing 10 μl of 1 μg/ml BSA were used for positive controls. The plate was then read at an optical density of 620 nm. Protein levels were calculated using a standard curve based on the absorbance of BSA.

#### Biochemical data analysis

The absorbance readings from replicate wells were converted into mean values, which were then divided by the corresponding protein values to determine each mosquito’s enzyme activity. Standard curves were constructed for each enzyme assay using BSA for protein quantification and cytochrome C, α/β-naphthol, and CDNB standards for MFO, EST, and GST assays, respectively. Calibration curves were linear (*R*^2^ > 0.98) across the tested concentration ranges. To ensure assay consistency, intra- and inter-assay variation was calculated from triplicate positive controls, with the coefficient of variation (CV) remaining below 10% across all plates. Plate optical path-length corrections were applied by normalizing absorbance to a path length of 0.6 cm, as measured for 96-well microplates, to enable accurate conversion of absorbance to molar concentration using Beer’s law.

All enzyme activity data were first tested for normality (Shapiro–Wilk) and homogeneity of variance (Levene’s test). The data were log-transformed and subjected to a normality test before analysis in IBM SPSS Statistics version 28.0 to fulfill the assumptions of ANOVA. A one-way ANOVA was carried out to determine whether the AChE activity of *Ae. aegypti* strains after inhibition with propoxur, monooxygenases, EST, and GSTs differed significantly from one another (Welch’s post hoc test, *P* < 0.05).

## Results

### Susceptibility to pyrethroids and organophosphates

**Fig. 2:**
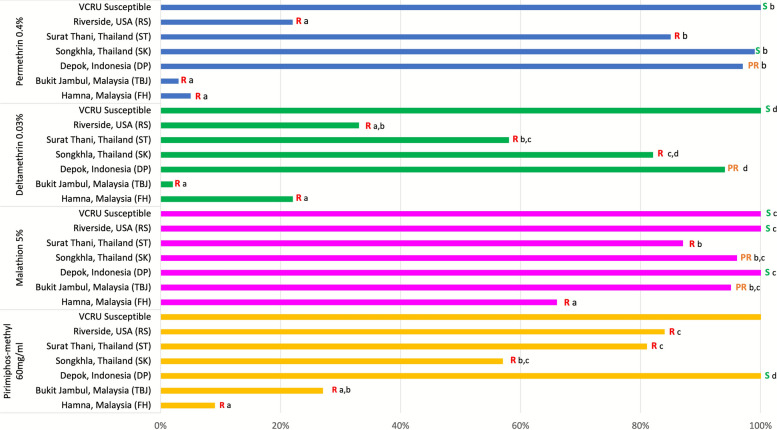
The susceptibility status of *Ae. aegypti* in different localities to the pyrethroid and organophosphate insecticides. The different letters represent the significant values of one-way ANOVA at *P* < 0.05 between localities for each insecticide. R = resistance, PR = possible resistance, S = susceptible

The Malaysian *Aedes aegypti* of both FH and TBJ strains showed significantly high phenotypic resistance toward PY and OP insecticides (mortality of 9–22%; one-way ANOVA, *P* < 0.05) compared to other localities, except possible resistance for TBJ exposed to malathion (mortality 95%; Fig. 2). The RS strain from the USA also exhibited high phenotypic resistance to PY and was not significantly different from the Malaysian strain (mortality 22–33%; one-way ANOVA, *P* < 0.05) but remained susceptible to malathion. *Aedes aegypti* collected from ST, Thailand, showed phenotypic resistance to PY and OP (mortality 58–87%). However, the SK strain, also from Thailand, remained susceptible to PY (mortality rate 99%), but not to the OP group. Mosquitoes from Indonesia were found to be less resistant to PY, and susceptible to OP (Fig. 2).

### Detection of target-site *kdr* mutation at domain II and III of VGSC

**Fig. 3:**
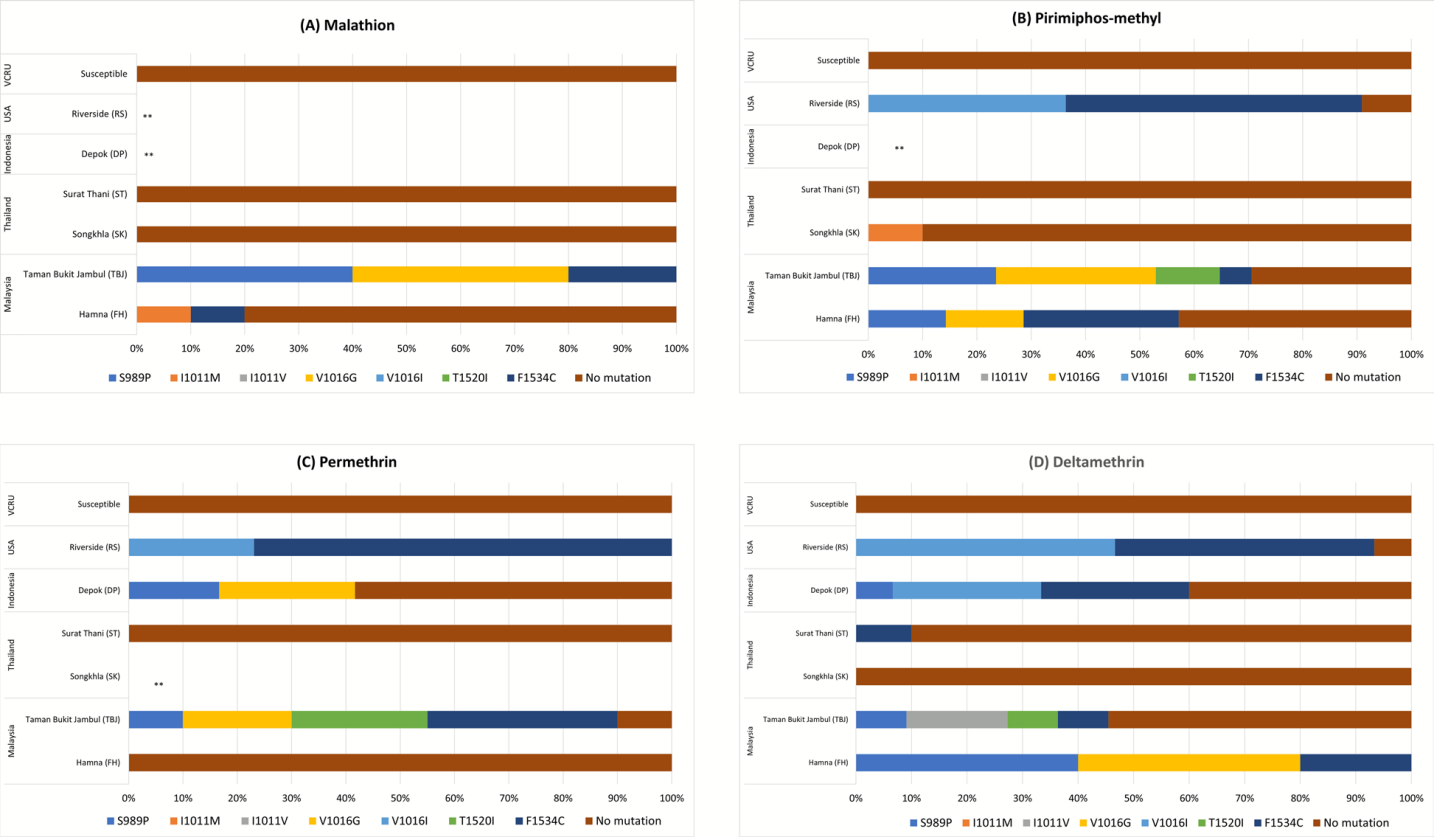
Point mutations of domains II and III in the voltage-gated sodium channel found in *Ae. aegypti* collected from Malaysia, Thailand, Indonesia, and the USA after exposure to **A** 5% malathion, **B** 60 mg/m^2^ pirimiphos-methyl, **C** 0.4% permethrin, and **D** 0.03% deltamethrin. The VCRU susceptible strain (dead individual) was confirmed not to carry any known *kdr* mutations. **Mutation analysis was not conducted on susceptible mosquitoes due to no survivors

The VGSC resistance alleles were detected in insecticide-resistant mosquitoes from the DIIS6 region (codons 989, 1007, 1011, 1016) and the DIII6 region (codons 1520 and 1534) (Fig. 3). Resistant mosquitoes from Thailand had a lower frequency of resistance alleles, with 10% I1011M frequency in SK-pirimiphos-methyl-resistant and 10% F1534C frequency in ST-deltamethrin-resistant samples. The changes at codon position 1016 from isoleucine (ATA) to methionine (ATG) and codon position 1534 from phenylalanine (TTC) to cysteine (TGC) were discovered in pirimiphos-methyl-resistant, permethrin-resistant, and deltamethrin-resistant mosquitoes of the RS USA strain. Surprisingly, the V1016I mutation was also detected in DP Indonesia for PY-possible-resistant individuals.

A mixture of *kdr* mutation alleles was found in both Malaysian strains. Pirimiphos-methyl-resistant and permethrin-resistant TBJ exhibited four resistance codons: S989P, V1016G at domain II, and T1520I and F1534C for domain III (Fig. 3b, c, and d). Interestingly, this is the first detection of T1520I in Malaysia, which was reported in India earlier [35]. At codon position 1520, the wild-type amino acid threonine (ACC) changes to isoleucine (ATC) due to a C-to-T substitution. Our sequencing also detected I1011V at a frequency of 15% in deltamethrin-resistant mosquitoes. Meanwhile, only three mutations were detected in malathion-resistant TBJ mosquitoes, which were S989P, V1016G, and F1534C. The same mutations were detected in the FH strain for pirimiphos-methyl-resistant and deltamethrin-resistant mosquitoes. In the malathion-resistant FH strain, changes at position 1011, from isoleucine (ATA) to methionine (ATG), were also detected. No mutation was detected at A1007G in the DIIS6 region in any sample.

### Distribution of *kdr* mutations and multiple loci of the genotypes

We identified eight substitution combinations, comprising six double-locus, one triple-locus, and one quadruple-locus, from four countries genotyped for DIIS6 and DIIIS6 (Table 1). The locus genotype with a combination of two amino acid substitutions of V1016I+F1534C (Type 5) was found in pirimiphos-methyl-resistant, permethrin-resistant, and deltamethrin-resistant USA samples. We observed that the presence of these substitution patterns led to high resistance to both PY and OP insecticides.

Meanwhile, type 6 (T1520+F1534C-homozygote) was detected only in permethrin-resistant TBJ, Malaysia. Quadruple-locus pirimiphos-resistant homozygote, S989+V1016+T1520I+F1534 (Type 8), and triple-locus malathion-resistant homozygote, S989+V1016+F1534 (Type 7), were also found in the same spot of TBJ strain from Malaysia. This also supports the finding of high resistance in phenotypic tests toward PY and OP insecticides. We observed that Malaysia and Indonesia exhibited the greatest occurrence of double-locus *kdr* type 2 (S989P+V1016G). Thailand samples had no multiple-locus patterns of substitution, and only one homozygous F1534C was detected among all samples from Surat Thani. This suggests that the phenotypic resistance was not led by the target-site *kdr* mutation. The VCRU susceptible strain was not analyzed because no survivors were observed in the insecticide exposure tests.

### Biochemical assays on enzymatic activity

**Fig. 4:**
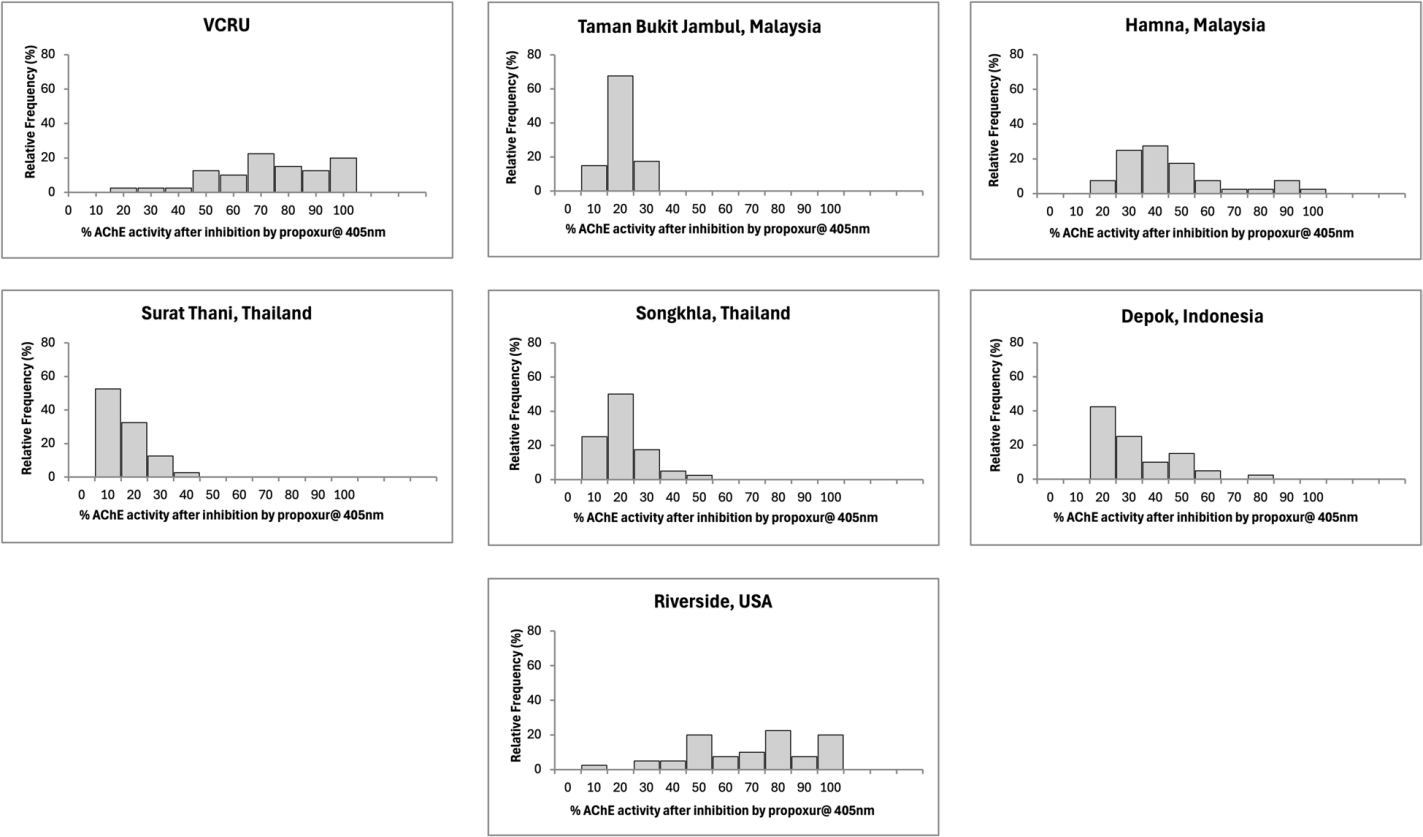
Distribution of remaining acetylcholinesterase activity after inhibition with propoxur in *Ae. aegypti* samples from seven strains (*n* = 40). Results are expressed as % AChE activity after inhibition by propoxur

**Fig. 5:**
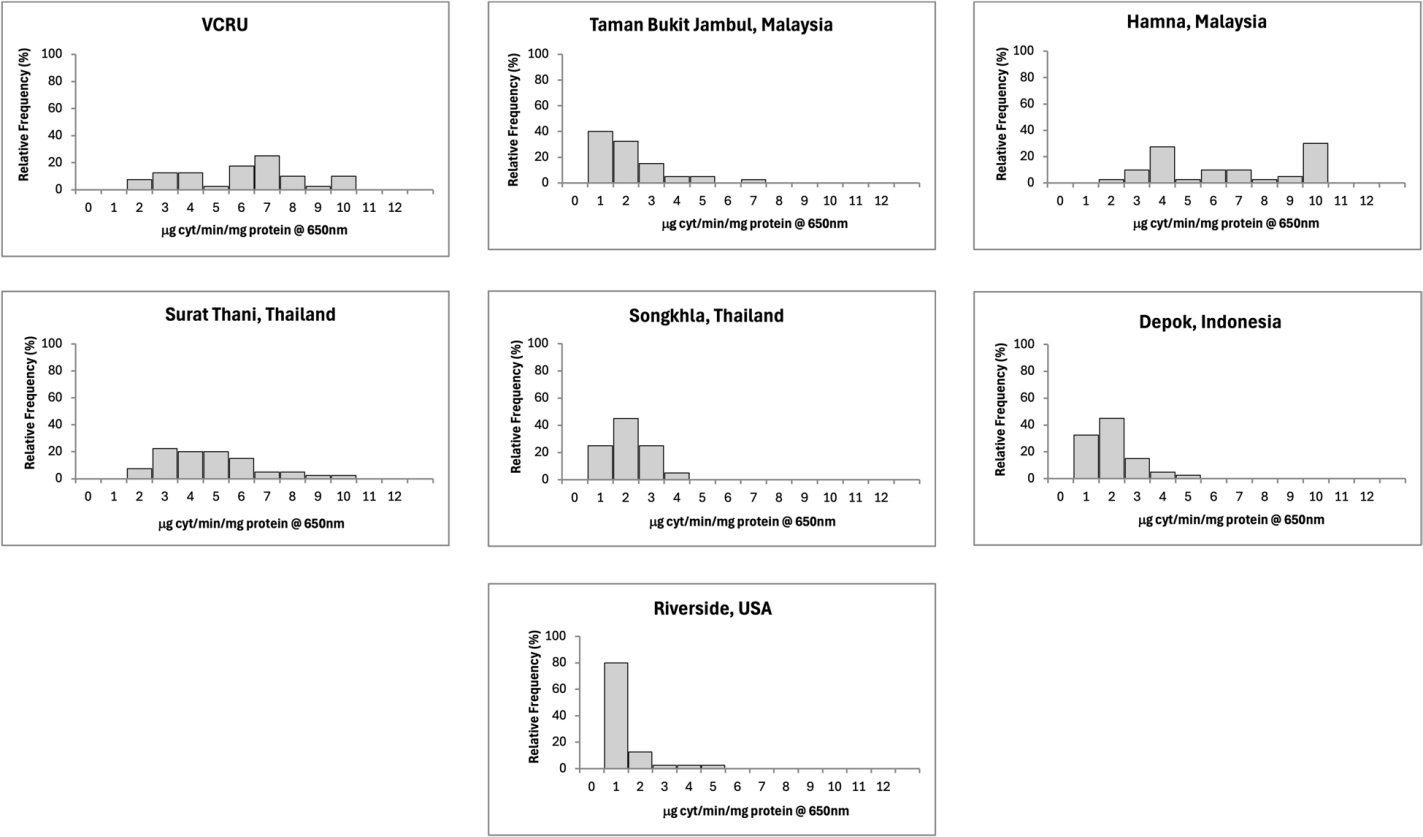
Distribution of mixed-function oxidase (MFO) from *Ae. aegypti* originating from seven localities (*n* = 40). Results are expressed in equivalent units of cytochrome P450 (μg/min/mg protein)

**Fig. 6:**
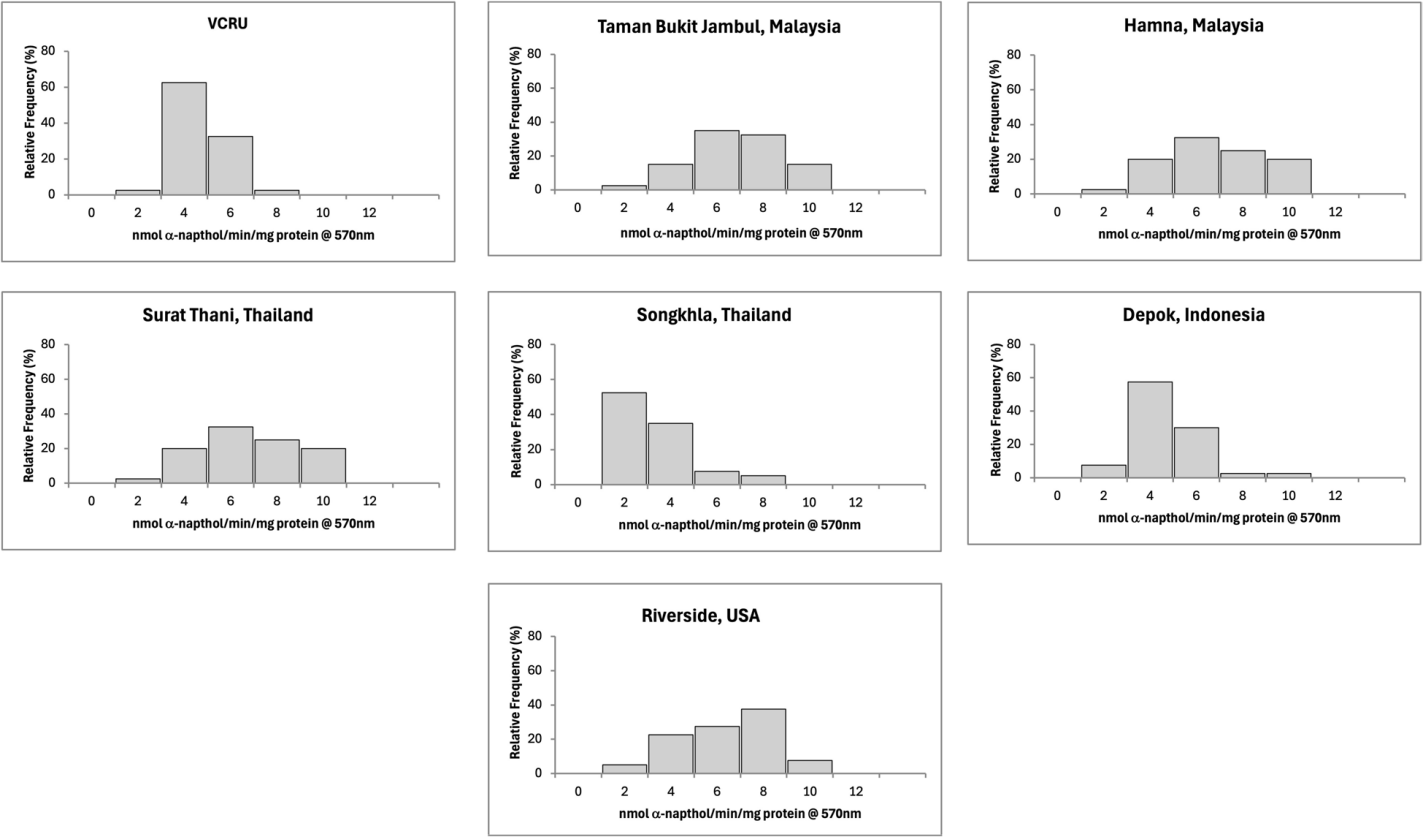
Distribution of α-esterase in seven localities for *Ae. aegypti* mosquitoes (*n* = 40). Results are expressed in nmol α-naphthol/min/mg protein

**Fig. 7:**
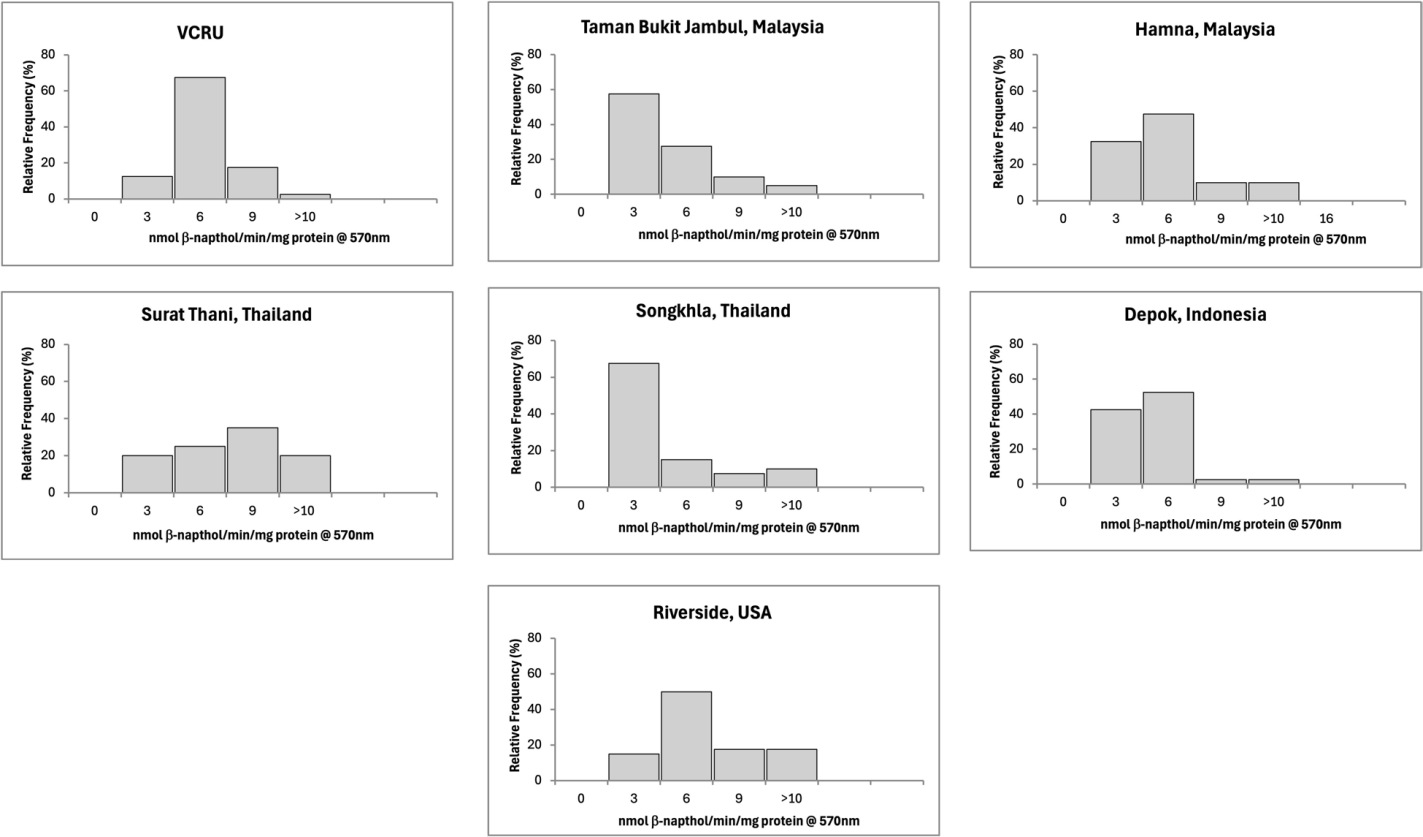
Distribution of β-esterase from *Ae. aegypti* mosquitoes collected from different countries (*n* = 40). Results are expressed in nmol β-naphthol/min/mg protein

**Fig. 8:**
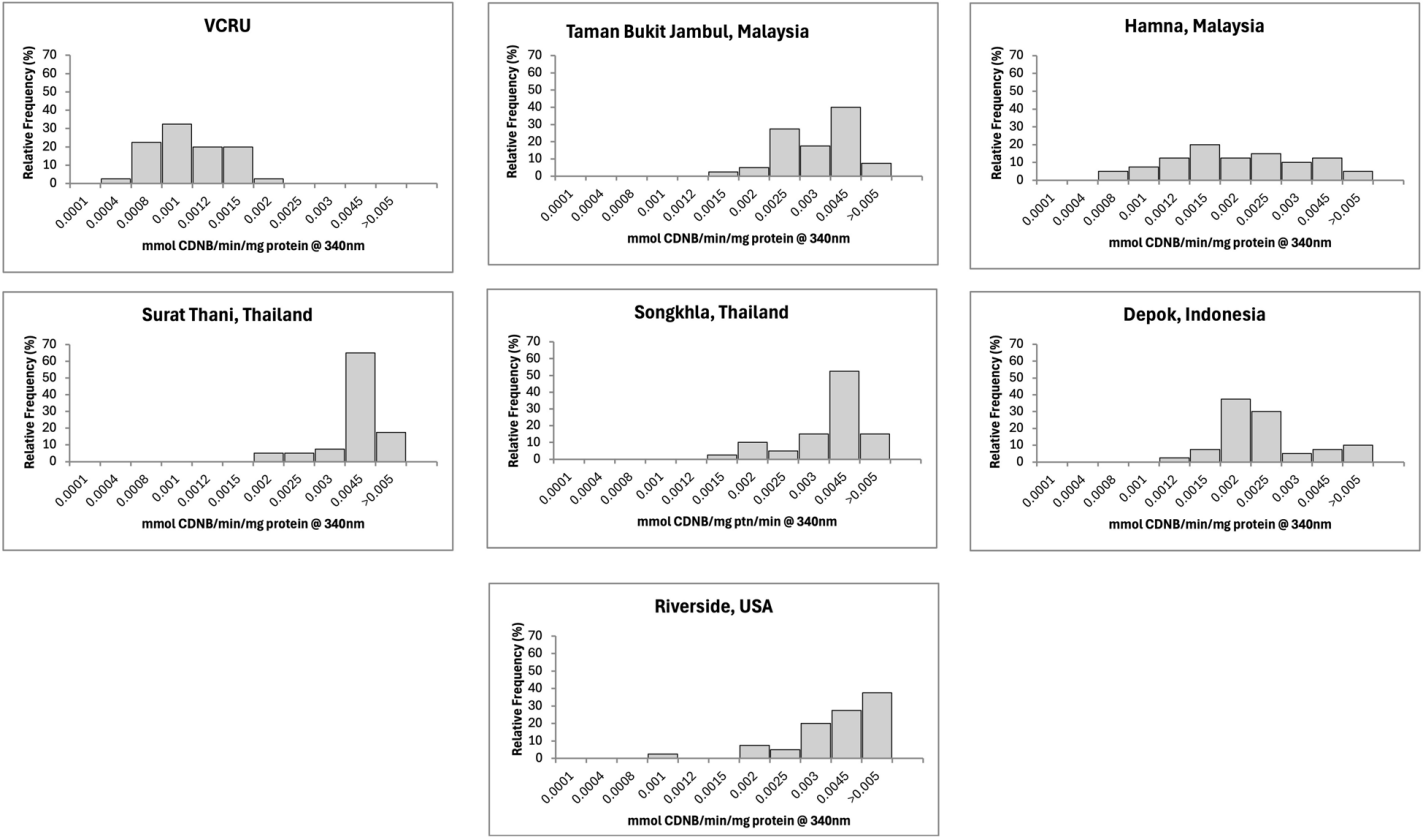
Distribution of glutathione S-transferase (GST) in *Ae. aegypti* originating from different countries in Asia and the USA (*n* = 40). Results are expressed in mmol CDNB/min/mg protein

The mean enzymatic activity of each strain is reported in Table 2, and the frequency distributions of enzymes are shown in Figs. 4, 5, 6, 7, and 8 for the remaining activity of AChE after inhibition with propoxur, MFO, α-EST, β-EST, and GST assays. The RS USA strain exhibited a nine- to 10-fold increase in α-EST and β-EST activity and a three- to fivefold increase in MFOs relative to the VCRU susceptible reference strain (Table 2; Welch’s ANOVA, *P* < 0.05). In contrast, GST activity in the RS strain was significantly lower than that of VCRU (Welch’s ANOVA, *P* < 0.05), suggesting a distinct metabolic profile in which ESTs and oxidases, rather than GSTs, play a dominant role in detoxification. This frequency was well distributed and skewed to the right side, indicating that resistance had occurred at the metabolic sites (Figs. 4, 5, 6, 7, 8), thus including a high frequency of *kdr* mutations (Fig. 3 and Table 1). The SK strain also showed an increasing pattern in all enzymes, but only the remaining AChE after inhibition with propoxur and GST showed significant values (Welch’s ANOVA, *P* < 0.05; Table 2). Meanwhile, lower frequency of *kdr* mutations was detected in the samples from SK (Table 1). The DP strain from Indonesia was found to be susceptible to OP and possibly resistant to PY (Fig. 2). These results were supported by the metabolic enzymes, where the upregulation of α-EST and GST was not significantly different from that of the VCRU reference strain (Welch’s ANOVA, *P* < 0.05). Malaysia strains from both TBJ and FH showed high resistance to insecticides (Fig. 2), with the presence of multiple *kdr* mutations (Table 1), indicating upregulation of α-EST, β-EST, and GST enzymes as a mechanism of resistance for the FH strain. Still, it did not differ significantly from the VCRU reference strain (Welch’s ANOVA, *P* > 0.05).

**Table 2:** Mean remaining activity of acetylcholinesterase (AChE), mixed function oxidase (MFO), esterases, and glutathione S-transferase (GST) for *Ae. aegypti* mosquitoes from different countries (*n* = 40)

Country	Strain	AChE^1^	MFO^2^	α-EST^3^	β-EST^4^	GST^5^
		Mean ± SE	Mean ± SE	Mean ± SE	Mean ± SE	Mean ± SE
Reference (susceptible)	VCRU	16.91 ± 1.24^a^	10.44 ± 0.84^a^	0.39 ± 0.02^a^	0.56 ± 0.03^a^	3.36 × 10^−4^ ± 0.00^a^
Malaysia	Taman Bukit Jambul (TBJ)	5.46 ± 2.45^c^	4.24 ± 1.34^b^	0.99 ± 0.31^b^	0.82 ± 0.36^a^	3.74 × 10^−3^ ± 0.00^a,b^
	Hamna (FH)	14.04 ± 1.88^a,b^	24.85 ± 6.51^a^	0.59 ± 0.14^a,b^	0.88 ± 0.24^a^	9.64 × 10^−4^ ± 0.00^a^
Thailand	Surat Thani (ST)	2.69 ± 0.40^c^	11.64 ± 0.65^a^	0.69 ± 0.05^a,b^	0.81 ± 0.07^a^	1.37 × 10^−3^ ± 0.00^a,b^
	Songkhla (SK)	16.92 ± 7.49^b^	33.70 ± 12.51^a^	1.06 ± 0.64^a,b^	2.34 ± 1.15^a^	3.49 × 10^−3^ ± 0.00^b^
Indonesia	Depok (DP)	8.92 ± 2.14^b,c^	3.10 ± 0.49^b^	0.43 ± 0.04^a,b^	0.44 ± 0.05^a^	9.16 × 10^−4^ ± 0.00^a^
USA	Riverside (RS)	126.21 ± 19.10^d^	24.36 ± 5.36^a^	1.45 ± 0.16^c^	6.03 ± 0.70^b^	3.61 × 10^−3^ ± 0.00^b^

^1^Expressed in % AChE activity after inhibition by propoxur

^2^Expressed in equivalent units of cytochrome P450 (μg/min/mg protein)

^3^Expressed in nmol α-naphthol/min/mg protein

^4^Expressed in nmol β-naphthol/min/mg protein

^5^Expressed in mmol CDNB/min/mg protein

## Discussion

Understanding the mechanisms behind insecticide resistance in *Ae. aegypti* is crucial for developing sustainable vector control strategies. Resistance can result from multiple mechanisms, including increased metabolic detoxification and target-site mutations in the mosquito’s nervous system. These mechanisms may act independently or together, causing different levels of resistance across populations and regions. Our study found high phenotypic resistance of *Ae. aegypti* to PYs (permethrin and deltamethrin) and OPs (malathion and pirimiphos-methyl) in strains from PN, Malaysia, and RS, USA. In contrast, the DP Indonesia strain was susceptible to OPs and only weakly resistant to PYs. Interestingly, Thai strains (SK and ST) showed strong resistance to PYs, despite not having detectable *kdr* mutations. This suggests that phenotypic resistance may be linked to other physiological or environmental factors less influenced by genetic markers.

Several elements, including insecticide resistance, environmental adaptation, and human activities, play critical roles in shaping this phenomenon [36]. The surge in cases highlights the critical importance of understanding the dynamics influencing the resilience of *Ae. aegypti* in both countries. The intensive use of insecticides for vector control has inadvertently exerted selective pressure on mosquito populations, favoring individuals carrying alleles associated with resistance [22]. The escalating resistance to PY and OP insecticides presents a complex challenge rooted in distinct agricultural practices and environmental conditions. For example, the same insecticides used to control agricultural pests are employed in vector control programs for diseases like malaria and dengue [37]. Pests exposed to insecticides in one sector (e.g., agriculture) may develop resistance mechanisms that confer cross-resistance to insecticides used in the other sector (e.g., public health). Pests often develop resistance to multiple insecticide classes through various mechanisms, including target-site modifications, enzymatic detoxification, and reduced penetration or excretion of insecticides [38].

New detections of the T1520I mutation, which has never been reported in Malaysia, as well as the I1011M mutation, were found in this study. The T1520I mutation in *Ae. aegypti* is part of a broader context of knockdown resistance (*kdr*) mutations that are associated with insecticide resistance, particularly against PY and DDT, and was first reported in Delhi [35, 39]. The presence of T1520I, although less prevalent than other mutations, plays a role in enhancing resistance when combined with F1534C, which is linked to permethrin resistance but does not affect sensitivity to deltamethrin [40]. Our results revealed a high frequency of the T1520I+F1534C dual-locus mutation in the TBJ strain from Malaysia (Table 1). The distribution of these mutations varies across regions, with studies indicating a lack of recombination among haplogroups in Indian populations, suggesting stable resistance mechanisms [41]. The I1011M *kdr* mutation in *Ae. aegypti* is less prevalent in Asia and was only detected at a low level in malathion-resistant FH Malaysia strains. This mutation has been formally reported in Thailand, Vietnam, Brazil, French Guyana, and Martinique [42, 43].

Regional variation was observed with the S989P, V1016G, and F1534C mutations which have dominated Southeast Asian populations and are all associated with PY resistance. In contrast, V1016I was prevalent in the Americas but has now emerged in Asian strains. High frequencies of F1534C and V1016G have been widely reported in *Ae. aegypti* and are associated with PY resistance [26, 27]. In our study, the homozygous V1016I mutation was detected in the pirimiphos-resistant and deltamethrin-resistant RS USA strain. Although V1016I is uncommon in *Ae. aegypti* populations across Asia, where the V1016G mutation predominates, it was found in the DP strain from Indonesia among individuals classified as possibly PY-resistant. Such findings underscore the global dissemination and regional adaptation of resistance alleles. As reviewed by Uemura et al. [44], V1016I resistance mutations have been found in Argentina, Brazil, Burkina Faso, Colombia, Ghana, Iran, the USA, and Venezuela (since 2023). The mutation occurred at DII6 for codons 1016, suggesting that the dissemination of resistance has transitioned from the Americas to Asia. This mutation, along with others in the VGSC gene, has been associated with a substantial reduction in PY binding and efficacy of PY insecticides, which are commonly used for vector control.

The V1016I mutation may not autonomously diminish PY sensitivity; however, it could engage in a synergistic interaction with other knockdown resistance mutations, such as F1534C or V410L, which can lead to resistance levels varying from 3.9- to 56-fold depending on the specific PY used [45]. In PY-resistant *Ae. aegypti* populations, V1016I is often linked to another mutation, F1534C, which confers sodium channel resistance only to type I PYs, including permethrin, but not to type II PYs, including deltamethrin [46]. Mosquitoes carrying both V1016G and F1534C were found to exhibit greater PY resistance than those carrying F1534C alone [40]. *Aedes aegypti* mosquitoes with a high frequency of double-homozygous resistant genotypes (II/CC) contributed to increased survivorship of mosquitoes at varying distances from insecticide application sites [47]. In our study, this interaction implies that V1016I or V1016G could be integral in augmenting mosquitoes’ comprehensive resistance profile when conjoined with F1534C, resulting in resistance to permethrin and deltamethrin. The emergence of V1016I in parts of Asia mirrors dominant V1016I–F1534C haplotypes long documented across Latin America, where these combinations track strong operational resistance [48, 49].

Multiple knockdown resistance (*kdr*) mutations in the VGSC gene were identified. The V1016G, S989P, and F1534C mutations are typically associated with the Indo-Pacific region [50]. The presence and frequency of these mutations across various populations can provide insights into the potential for resistance development similar to that found in this study in Malaysia and Indonesia. This mutation, when present with other *kdr* mutations, significantly enhances the survival of mosquitoes under insecticide exposure [51, 52]. Specific haplotypes, particularly those containing multiple *kdr* mutations, are associated with higher resistance levels, emphasizing the need for targeted monitoring [53]. This mutation enhances resistance levels, impacting the effectiveness of insecticides and complicating vector control efforts against dengue and other viral transmissions [44]. For instance, the combination of V1016G and F1534C can confer up to 1100-fold increased resistance to PY [41]. Research has highlighted V1016G, S989P, and F1269C mutations associated with PY resistance in Southeast Asia, particularly Singapore and Indonesia [54]. In Vietnam and Cambodia, high frequencies of V1016G as well as L982W and F1534C mutations have been reported, which are associated with high levels of PY resistance, indicating a concerning trend of increasing resistance [55].

Beyond *kdr* mutations, other resistance mechanisms may also play a role, indicating that reliance solely on *kdr* mutation monitoring may not capture the complete picture of resistance dynamics [56]. OP resistance is less frequently associated with genetic mutations, making it less likely to develop in insect populations [57]. The mode of action primarily involves the inhibition of AChE, leading to an accumulation of acetylcholine at nerve synapses [58, 59]. In this study, metabolic detoxification, particularly through the overexpression of cytochrome P450 monooxygenases, ESTs, and GST, may contribute significantly to insecticide resistance. In the RS USA strain, the activity of ESTs, GSTs, and MFOs was increased between three- and 100-fold compared to the VCRU reference strain. The co-occurrence of metabolic resistance and multiple *kdr* mutations may explain its resistance in the PY bioassays (Fig. 2). Resistance is most severe when metabolic and site mutation mechanisms co-occur. For example, the RS strain exhibited both high enzymatic activity and multiple *kdr* mutations, resulting in strong resistance to both PYs and OPs. Similarly, dual-locus mutations such as T1520I+F1534C or V1016G+F1534C conferred markedly higher resistance levels than single mutations. This interplay highlights the complexity of resistance evolution and the challenge of relying on insecticides with a single mode of action [60].

In contrast, several strains appeared to have only a partial set of the screened resistance mechanisms. The Malaysian FH strain displayed high resistance without notable upregulation of detoxification activity, suggesting that its resistance was more closely associated with target-site mutations than with metabolic mechanisms. In contrast, the TBJ Malaysia strain showed increased MFO activity alone. According to Rubio-Palis et al. [61], elevated metabolic enzyme activity, such as MFOs and GSTs, plays a significant role in resistance. In *Ae. aegypti*, while *kdr* mutations increased significantly after deltamethrin selection, only α-EST activity remained elevated, indicating a complex relationship between mutations and enzyme upregulation [62]. While PY faces significant resistance challenges, the development of OP resistance remains limited. This suggests that the mechanisms and evolutionary pressures differ markedly between these two classes of insecticides.

In addition, despite the phenotypic resistance exhibited by the Thailand strains (SK and ST) to deltamethrin exposure, they showed limited frequency of *kdr* mutations. In *Ae. aegypti* from Côte d’Ivoire, phenotypic resistance was observed against several insecticides, with mortality rates varying significantly across sites, yet no *kdr* mutations were detected [63]. Thus, the upregulation of the remaining AChE following propoxur inhibition and GST might be associated with the resistance status of the Thai strains. Furthermore, the moderate resistance observed in the ST strain (54–64% mortality), despite the absence of *kdr* mutations and a lack of significant elevation in detoxification enzyme activity, indicates that alternative mechanisms may contribute to the observed PY tolerance. Such discrepancies in susceptibility could arise from behavioral avoidance of insecticide contact, reduced insecticide penetration due to cuticular thickening or compositional changes, or other molecular processes not captured by the current assays, such as the upregulation of ion channel modulators or efflux transporters. The DP Indonesian strain showed high susceptibility to OP and incipient resistance to PY, as indicated by their phenotypic resistance. Depok, Indonesia, is a suburban extension of Jakarta and a rapidly growing urban area that has yet to experience less insecticide pressure, allowing for a more susceptible mosquito population. However, the growing urban area may lead to more resistant individuals due to the selective pressures of insecticides [64] and habitat availability, which is increasingly at risk of vector-borne diseases that require management effort using insecticides as *Ae. aegypti* expands its range [65]. The Indonesian DP and the US RS strains remain susceptible to malathion. This may be due to a lack of prior selection or conflicting metabolic pathways involved in malathion detoxification. Malathion undergoes P450-mediated bioactivation to its more toxic metabolite, malaoxon [66]. However, P450s are readily enhanced in resistant insects, leading to susceptibility to OPs that undergo oxidative desulfurization despite resistance to other insecticides. Nonetheless, this dynamic is poorly understood and may not always affect resistance, as shown in various studies where mosquitoes remained susceptible despite enzyme overexpression [67, 68].

Our biochemical assay results are consistent with regional reports from Southeast Asia and broader global syntheses, which show that metabolic detoxification frequently accompanies or amplifies PY resistance in *Ae. aegypti.* Reviews and WHO regional assessments document widespread elevation of MFO/P450, EST, and GST activity in urban *Ae. aegypti* populations, often linked to intense insecticide use and household aerosols/space sprays, with *Ae. albopictus* typically showing lower urban selection pressure by comparison [69]. The combined presence of multiple *kdr* mutations and elevated metabolic enzyme activity has significant operational implications for vector control programs. Populations exhibiting both target-site mutations (e.g., V1016G/I, F1534C, and T1520I) and metabolic resistance markers such as elevated cytochrome P450, EST, and GST are more likely to withstand standard PY-based interventions [69, 70]. This dual resistance mechanism reduces the efficacy of PY-based household aerosols, ultralow-volume fogging, and indoor residual spraying, which remain central to dengue control programs across Southeast Asia [31, 48]. Operationally, this implies that PY-based interventions alone are insufficient in regions where these combined mechanisms are prevalent. Rotational use of insecticides from different classes, incorporation of synergist-based formulations (e.g., piperonyl butoxide [PBO]), and regular molecular–biochemical surveillance are therefore essential to sustaining control efficacy and delaying further resistance selection [69].

## Conclusions

Our results demonstrate widespread resistance to PYs and/or OPs in *Ae. aegypti* populations from Malaysia, Thailand, and the USA. Both *kdr* mutations and elevated metabolic enzyme activity were observed, highlighting the need for integrated resistance management strategies**.** Future studies should validate metabolic mechanisms using synergist (PBO) bioassays and gene expression analysis. Although these assays were not included in this study, the biochemical assays provided indirect evidence of PBO-inhibitable monooxygenase-mediated resistance. Future work will incorporate PBO and reverse transcription quantitative PCR (RT-qPCR) analyses to confirm the involvement of specific cytochrome P450 and GST genes.

## Data Availability

Data supporting the main conclusions of this study are included in the manuscript.
